# Linking composition and function: a two-year metatranscriptomic study of microbial adaptation in an industrial wastewater treatment plant

**DOI:** 10.1093/femsec/fiag051

**Published:** 2026-05-18

**Authors:** Asala Mahajna, Ranko Gacesa, Karel J Keesman, Gert-Jan W Euverink, Andreas Froemelt, Carlos J Melián, Bayu Jayawardhana

**Affiliations:** Engineering and Technology Institute Groningen, Faculty of Science and Engineering, University of Groningen, Nijenborgh 4, 9747 AG Groningen, The Netherlands; Department of Genetics, University of Groningen and University Medical Center Groningen, Antonius Deusinglaan 1, 9713 AV Groningen, The Netherlands; Department of Gastroenterology and Hepatology, University of Groningen and University Medical Center Groningen, Antonius Deusinglaan 1, 9713 AV Groningen, The Netherlands; Mathematical and Statistical Methods – Biometris, Wageningen University, Radix, building 107, Droevendaalsesteeg 1, 6708 PB Wageningen, The Netherlands; Engineering and Technology Institute Groningen, Faculty of Science and Engineering, University of Groningen, Nijenborgh 4, 9747 AG Groningen, The Netherlands; Eawag, Swiss Federal Institute of Aquatic Science and Technology, Überlandstrasse 133, 8600 Dübendorf, Switzerland; Department of Fish Ecology and Evolution, Swiss Federal Institute of Aquatic Science and Technology, Seestrasse 79, CH-6047 Kastanienbaum, Switzerland; Department of Aquatic Ecology, Institute of Ecology and Evolution, University of Bern, Bern 3012, Switzerland; Engineering and Technology Institute Groningen, Faculty of Science and Engineering, University of Groningen, Nijenborgh 4, 9747 AG Groningen, The Netherlands

**Keywords:** activated sludge, biodiversity-ecosystem functioning, complementarity and redundancy, environmental filtering, saline industrial wastewater, metatranscriptomes

## Abstract

Industrial saline wastewater presents a major challenge due to high salinity. While most previous studies focus on domestic activated sludge processes using 16S rRNA sequencing, this study provides metatranscriptomic insight into an industrial saline wastewater treatment plant in the Netherlands operating a facultative activated sludge process. The process, characterized by alternating aeration and non-aeration phases, generates dynamic redox conditions that drive microbial succession and adaptive metabolic responses over time, in contrast to conventional activated sludge processes that typically operate under more stable aeration regimes and comparatively steady redox conditions. Over two years and four months, 32 metatranscriptomic profiles were generated alongside comprehensive physicochemical and operational measurements. The activated sludge harbored a diverse, metabolically active community contributing to nitrogen, carbon, and phosphorus removal, as annotated via SEED Subsystems. Functional gene richness saturated more rapidly than taxonomic richness, reflecting substantial functional redundancy, while complementarity effects under lower-diversity conditions suggested cooperative interactions that enhance ecosystem functionality. Notably, Thermodesulfobacteriota, including sulfur-reducing Desulfobulbia, emerged as a core and environmentally filtered group under saline conditions. This study demonstrates that longitudinal metatranscriptomic profiling can capture temporally resolved functional responses in industrial microbial communities, offering insight into microbial resilience, adaptation, and the ecological principles underpinning engineered bioremediation systems.

## Teaser

In an industrial saline wastewater plant, microbial communities dynamically adjust their functional activity under alternating aeration, with cooperative interactions and core Thermodesulfobacteriota sustaining resilient ecosystem functioning.

## Background & summary

The sustainable treatment of industrial wastewater is critical to global environmental management and the achievement of circular economy goals (Shah 2023). Industrial effluents often contain complex mixtures of organic pollutants, heavy metals, and salts, posing significant challenges for conventional wastewater treatment technologies (Shah 2022, Shah 2024). Activated sludge systems remain the backbone of biological wastewater treatment, yet the microbiome dynamics within industrial wastewater treatment plants (WWTPs) are less understood compared to domestic systems (Mahajna et al. 2025). Given the increasing industrialization and the rising demand for effective bioremediation strategies, there is an urgent need to unravel the microbial ecology and functional potential of these specialized microbiomes (Lateef et al. 2023, Shah 2023).

Traditionally, microbial community studies in activated sludge have relied heavily on 16S rRNA gene amplicon sequencing, which offers taxonomic profiles primarily of bacterial populations (Wu et al. 2019, Mahajna et al. 2022). While 16S rRNA sequencing identifies bacterial taxa and can provide inferred functional potential, particularly with full-length reads, it does not measure gene expression and thus only indirectly reflects microbial activity (Handelsman 2008, Tedersoo et al. 2021). In contrast, metatranscriptomics directly captures the actively transcribed genes across bacteria, archaea, and eukaryotes, providing a real-time assessment of microbial function (Handelsman 2008, Tseng et al. 2015). This approach enables researchers to link the microbiome structure directly to microbiome function, offering insight into the dynamic ecosystem processes and microbial adaptations to environmental stresses (Maza-Márquez et al. 2022).

This study investigates the metabolically active microbial community and its functional dynamics within a full-scale industrial saline wastewater treatment plant in the Netherlands operating a facultative activated sludge process. We hypothesize that the alternating aeration and non-aeration phases generate fluctuating oxygen conditions that, together with the selective pressure of saline industrial influent, shape microbial succession and functional adaptation. Between 2014 and 2017, 32 metatranscriptomic samples were collected and integrated with comprehensive physicochemical and operational data, capturing the ribosomally active microbiome, including bacteria, archaea, and eukaryotes, and their expressed metabolic pathways. Using compositional analyses, we show that environmental filtering drives the emergence of specialized core taxa in the saline activated sludge microbiome. Cross-association analysis was applied to explore relationships between bacterial phyla and SEED subsystems functional categories, revealing structure-function relationships that support microbial ecosystem functioning. Additionally, species–function accumulation curves were used as a tool to unravel functional complementarity and redundancy in microbial communities.

## Methods

### Sampling campaign

**Figure 1:**
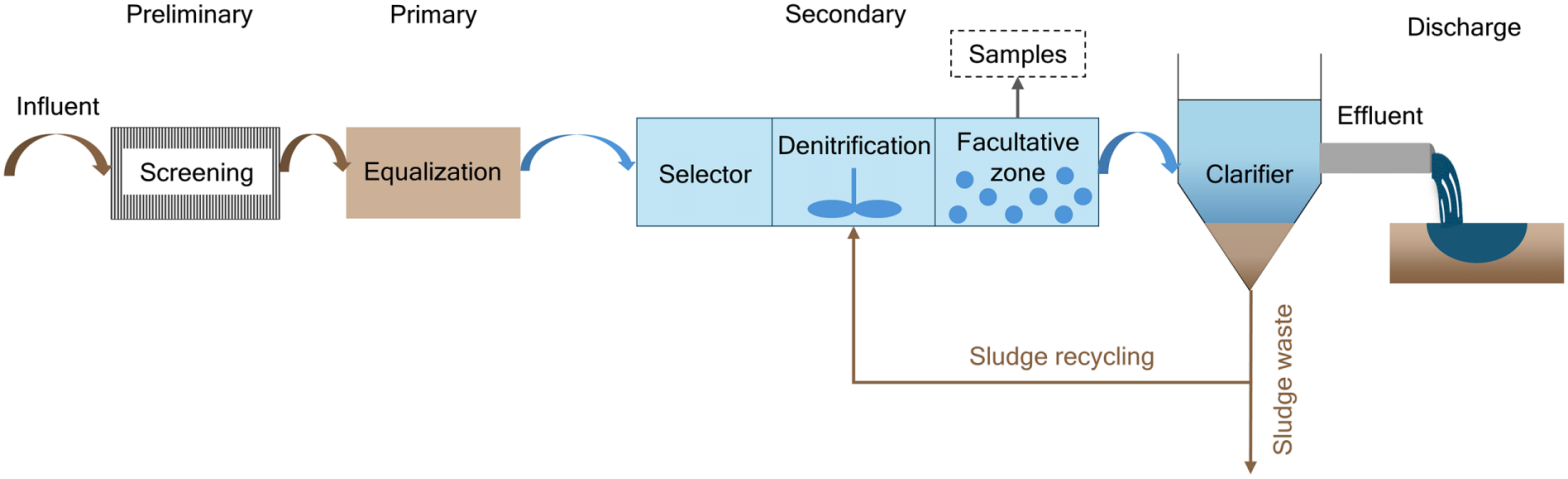
Schematic representation of the treatment process at Oosterhorn saline WWTP showing a preliminary treatment step based on screens, a primary treatment step based on an equalization tank, and a secondary treatment based on an activated sludge tank and a settling tank with sludge recycling (modified from Mahajna et al. 2025).

The sampling campaign was conducted in a wastewater treatment plant situated in the Oosterhorn industrial park near Delfzijl (The Netherlands), which processes industrial wastewater. It has a hydraulic capacity of 250 $\frac{{{{m}^3}}}{{\mathit{hour}}}$ and is capable of treating wastewater with a pollution load equivalent to 35 000 population equivalents (PE), with daily peaks of up to 60 000 PE. The treatment process is comprised of a series of steps starting with preliminary screening, followed by primary treatment in an equalization tank, and secondary treatment involving an activated sludge tank and a settling tank. The activated sludge tank is subdivided into three distinct zones: the selector, anoxic (denitrification), and facultative zones, with the latter’s air flow varying from 0 to 3 500 $\frac{{N{{m}^3}}}{h}$. Following treatment, the effluent is discharged into the Eems River, which eventually flows into the Wadden Sea (de Boks et al. 2009, North Water 2018). Part of the sludge from the settling tank is recycled to the secondary treatment zone. Figure 1 provides a schematic process flow diagram of the industrial wastewater treatment plant.

The sampling campaign, conducted between 2014 and 2017, yielded a comprehensive dataset from a full-scale industrial wastewater treatment plant in the Netherlands that treats saline industrial wastewater. The study focused on the facultative zone of the activated sludge process, collecting 32 meta-transcriptomic samples to investigate microbial metabolic activities and gene expression profiles (Mahajna 2024b,c). This genetic dataset is complemented by extensive physicochemical and biological measurements with full methodological details reported in the associated data publication (Mahajna 2024a, Mahajna et al. 2025).

### Metatranscriptome sequencing

A total of 32 activated sludge samples (8 mL each) were collected in 18 mL scintillation vials containing a stabilizing solution composed of 9.5 mL of 96% ethanol and 0.5 mL of 0.1 M sodium citrate (pH 4.2), prepared in-house as an ethanol-based RNA preservation solution. Ethanol inhibits RNase activity and rapidly fixes microbial cells, while the slightly acidic sodium citrate buffer further stabilizes RNA during storage (Rio et al. 2010, Ladell et al. 2019). This preservation solution maintained RNA integrity during storage at −20°C until further processing. For sample preparation, 1 mL aliquots were transferred to Lysing Matrix E vials (MP Biomedicals, Solon, OH, USA) and centrifuged at 10,000 × g for 10 min using an Eppendorf 5424 R centrifuge (Eppendorf AG, Hamburg, Germany) to concentrate microbial biomass.

RNA extraction was performed using a modified FastRNA ProSoil Direct Kit protocol (MP Biomedicals, Solon, OH, USA; Cat. No. 116070050), with modifications implemented to enable recovery of high-quality RNA suitable for downstream cDNA synthesis and metatranscriptomic sequencing. Briefly, 700 µL of RNApro Soil Lysis Solution was added to the sample pellet, followed by 350 µL of acidified phenol/chloroform. Samples were homogenized for 45 seconds using a FastPrep-24™ 5G bead-beating grinder (MP Biomedicals, Solon, OH, USA). An additional 600 µL of acidified phenol/chloroform was added, followed by centrifugation at 10,000 × g for 3 min at 4°C, after which the aqueous RNA-containing phase was recovered. RNA purification was performed using a spin-column protocol, followed by DNase treatment using RQ1 RNase-Free DNase (Promega, Madison, WI, USA; Cat. No. M6101) to remove residual genomic DNA. RNA was subsequently purified using the Zymo RNA Clean & Concentrator-5 Kit (Zymo Research, Irvine, CA, USA; Cat. No. R1015) and eluted in 30 µL nuclease-free water. RNA concentration was quantified using the Qubit™ RNA High Sensitivity (HS), Broad Range (BR), and Extended Range (XR) Assay Kits (Thermo Fisher Scientific, Waltham, MA, USA; Cat. No. Q32852).

Purified RNA was subsequently used for cDNA synthesis using the SensiFAST cDNA Synthesis Kit (Bioline, London, UK; Cat. No. BIO-65053) with random hexamer primers. Following first-strand synthesis, the second cDNA strand was synthesized using Klenow DNA polymerase (Promega, DNA Polymerase I Large (Klenow) Fragment; Cat. No. M2201), with random hexamer primers used for second-strand synthesis to generate double-stranded cDNA suitable for downstream library preparation and sequencing. The resulting cDNA was purified and eluted, and its concentration and quality were assessed using a Qubit Fluorometer (Thermo Fisher Scientific, Waltham, MA, USA) and the Quant-iT™ DNA High Sensitivity Assay Kit (Thermo Fisher Scientific; Cat. No. Q32851). Prior to library preparation, cDNA samples were diluted according to Nextera XT input requirements. Sequencing libraries were subsequently prepared using the Nextera XT DNA Sample Preparation Kit (Illumina, San Diego, CA, USA; Cat. No. FC-131-1024), and sequencing was performed on the MiSeq platform at the University Medical Center Groningen Sequencing Facility to generate metatranscriptomic datasets for downstream analyses.

### Bioinformatics pipeline

#### Profiling of ribosomally active community

Quality assessment of the raw sequence reads was performed using FastQC v0.12.1 (Andrews 2023) and MultiQC v1.22.2 (Ewels et al. 2016). The data underwent pre-processing and community profiling through Galaxy (Abueg et al. 2024), which included removing adapter sequences and trimming low-quality ends using Trimmomatic v0.39 (Bolger et al. 2014). A sliding window approach was applied, setting a quality threshold of 30 over 4 bases, with low-quality reads discarded. Taxonomic classification was performed using Kraken2 v2.1.3 (Wood and Salzberg 2014), referencing the RefSeq PlusPF database (O’Leary et al. 2016). Abundance was re-estimated with Bracken v3.0 (Jennifer et al. 2017). Total RNA-Seq data was utilized to capture the complete microbial community, ensuring the inclusion of taxa with high ribosomal RNA content, which could otherwise be missed.

#### Functional profiling

**Figure 2:**
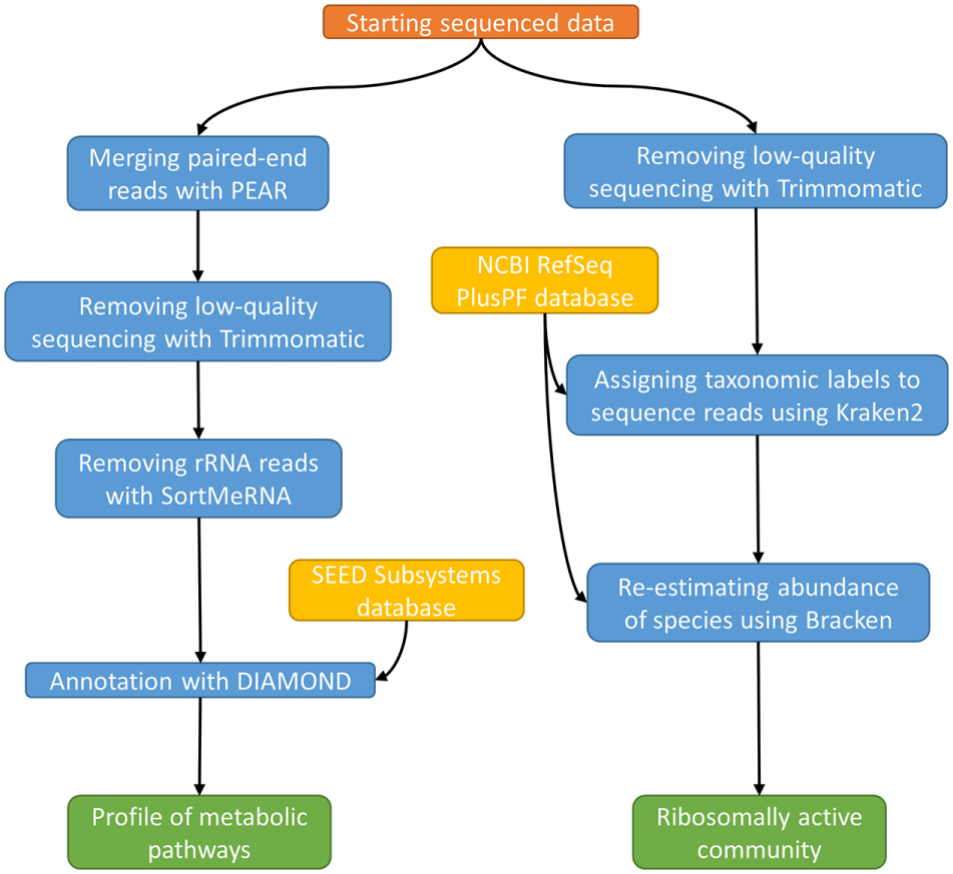
Bioinformatics pipeline for metatranscriptomic data analysis. The flowchart details two parallel computational pathways for processing starting sequenced data. The left path outlines the functional annotation process, beginning with the merging of paired-end reads using PEAR, followed by quality control with Trimmomatic, rRNA depletion via SortMeRNA, and protein annotation with DIAMOND against the SEED subsystems database to produce a profile of metabolic pathways. The right path describes the taxonomic classification process, utilizing Trimmomatic for quality filtering, Kraken2 for taxonomic labeling against the NCBI RefSeq PlusPF database, and Bracken for abundance re-estimation to characterize the ribosomally active community.

The functional profiling of the microbial community was carried out by first assessing the sequence quality using FastQC (Andrews 2023) v0.12.126 and MultiQC (Ewels et al. 2016) v1.22.2. Raw sequencing reads were processed through the SAMSA2 pipeline (Westreich et al. 2018), which employs PEAR (Jiajie et al. 2014) for merging paired-end reads, followed by adapter removal and quality trimming with Trimmomatic (Bolger et al. 2014). To focus on non-rRNA genes, rRNA sequences were excluded using SortMeRNA (Evguenia et al. 2012). Functional gene annotation was performed by aligning the processed sequences to SEED reference databases (Overbeek et al. 2014) using DIAMOND (Buchfink et al. 2021), providing a detailed functional profile for the 32 saline-activated sludge samples. This analysis identified key metabolic pathways and functional categories within the microbiome. The workflow of the bioinformatics pipeline designed and implemented to generate the microbiome data is outlined in Fig. 2.

### Technical validation

Comprehensive quality assurance was conducted at multiple stages to ensure the integrity of the metatranscriptomic data; details of the technical validation are found in the respective data descriptor paper (Mahajna et al. 2025). RNA and cDNA concentrations were quantified using Qubit™ assays following extraction and cDNA synthesis, respectively, and prior to library preparation. Post-sequencing, quality scores confirmed high base-call accuracy, with over 98% of bases achieving Phred scores ≥20. Read-level quality was consistent across samples, with forward reads generally exhibiting higher fidelity than reverse reads. FastQC v0.12.1 (Andrews 2023) and MultiQC v1.22.2 (Ewels et al. 2016) assessments further confirmed uniform sequencing performance, with consistent distribution patterns and minimal outlier influence. Rarefaction analysis demonstrated that sequencing depth was sufficient to capture both taxonomic and functional diversity, while feature accumulation curves confirmed comprehensive community representation across samples (Mahajna et al. 2025). Together, the validation steps establish the robustness and reliability of both the molecular and ecological dimensions of the dataset.

### Calculation of removal efficiencies

Removal efficiencies (RE) were calculated to quantify system-level treatment performance for biological oxygen demand (BOD), total nitrogen (TN), and phosphate in terms of phosphorus ($PO_4^{3 - }$). These metrics allow us to contextualize microbial community composition and functional activity in relation to the actual treatment outcomes of the activated sludge process.

Weekly averaged influent and effluent concentrations were used according to 1:


(1) \begin{eqnarray*}
R{{E}_i}\left( t \right) = \frac{{{{C}_{in,\, i}}\left( t \right) - {{C}_{out,i}}\left( t \right)}}{{{{C}_{in,i}}\left( t \right)}}{\mathrm{*}}100\, \forall i\left\{ {PO_4^{3 - },{\mathrm{TN}},{\mathrm{BOD}}} \right\},
\end{eqnarray*}

where ${{C}_{in,\, i}}( {\mathrm{t}} )$ and ${{C}_{out,\, i}}( {\mathrm{t}} )$are the weekly averaged concentrations of the *i*-th pollutant in the influent and effluent, respectively, at time *t*.

### Definition of Core Microbiome and Prevalence Score

To identify persistent taxa within the activated sludge microbiome, we defined the core community as all taxa present in 100% of samples (prevalence = 1).

The prevalence score of each taxon was calculated as the proportion of samples in which it was detected, ranging from 0 (absent in all samples) to 1 (present in all samples).

### Taxon-function correlation and clustering

Associations between microbial taxonomy and functional activity were explored by correlating phylum-level taxonomic abundances with SEED subsystem functional categories expression profiles. To account for the compositional nature of the data, abundances were CLR-transformed (centered log-ratio), projecting relative abundance data from the constrained simplex space into Euclidean space and mitigating spurious correlations. Spearman correlation, a non-parametric measure robust to non-normality and non-linearity, was computed to detect monotonic associations between taxa and functional pathways. Euclidean distance was used to quantify similarity between samples, and Ward’s hierarchical clustering was applied to group samples with similar taxonomic-functional relationships by minimizing within-cluster variance. This combined approach provides a general framework for revealing biologically and ecologically meaningful relationships between microbial taxa and their functional roles while appropriately handling compositional data.

## Results and discussion

### Ribosomally active microbial community as the driver of ecosystem multifunctionality

The wastewater treatment plant was operating under stable full-scale conditions throughout the 2-year and 4-month sampling campaign and was not in a start-up or commissioning phase. The system had already reached a mature operational stage prior to the beginning of the study. Metatranscriptomic analyses provide a comprehensive view of the microbial community in the activated sludge, shedding light on the ribosomally active members that drive the system’s capacity to remove carbon, nitrogen, and phosphorus (Tseng et al. 2015).

**Figure 3:**
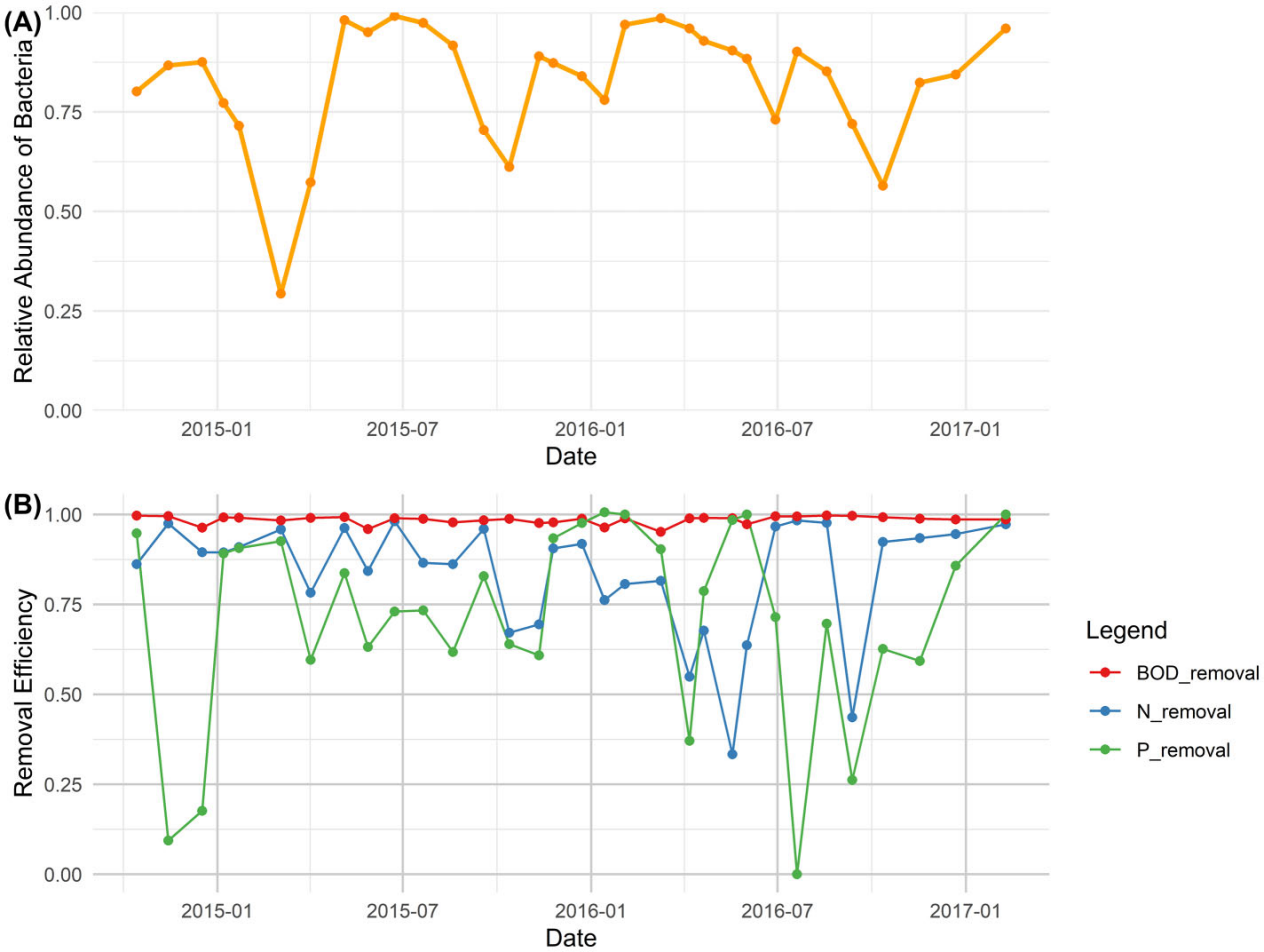
(A) Relative abundance of the bacterial community across 32 distinct ribosomally active microbial communities of the activated sludge. (B) Ecosystem functioning of the activated sludge process, as reflected by the removal efficiencies of nitrogenous (N_removal), carbonaceous (BOD_removal), and phosphorus (P_removal) pollutants across the same 32 samples.

Figure 3 (A) illustrates the relative abundance of the bacterial community within the ribosomally active microbial community of the activated sludge across 32 distinct samples. Bacteria constitute the dominant and functionally essential component of this engineered ecosystem, underpinning critical processes responsible for the removal of carbonaceous, nitrogenous, and phosphorus pollutants (Sui et al. 2024). Through aerobic and anaerobic respiration, heterotrophic bacteria degrade organic matter and reduce biochemical oxygen demand (BOD), a key indicator of carbonaceous pollution removal (Lyu et al. 2025). Nitrogen is removed via nitrification, carried out by autotrophic bacteria such as *Nitrosomonas* (phylum: Pseudomonadota) and *Nitrospira* (phylum: Nitrospirota), followed by heterotrophic denitrification, including pathways enabling simultaneous nitrification-denitrification under low-oxygen conditions (Su et al. 2025). Phosphorus removal is achieved predominantly through the activity of polyphosphate-accumulating organisms (PAOs), such as *Accumulibacter* (phylum: Pseudomonadota) and *Tetrasphaera* (phylum: Actinomycetota), which sequester excess phosphorus intracellularly during alternating anaerobic and aerobic phases (Albertsen et al. 2016, Roy et al. 2021). These microbial transformations collectively mediate the core ecosystem functions of nutrient cycling and pollutant removal, enabling the stable operation of activated sludge systems in compliance with environmental discharge standards (Tsang and Smith 2005).

Figure 3 (B) presents reactor performance by showing the respective removal efficiencies of BOD, total nitrogen (TN), and phosphate across the 32 samples. Variability in removal performance across samples reflects both temporal shifts in microbial community composition and the influence of dynamically changing environmental conditions within a single, continuously operated reactor.

During the monitoring period, decreases in phosphate (P) removal efficiency were observed (Fig. 3 B), coinciding with temporary reductions in the relative abundance of total bacteria (Fig. 3 A) and associated with episodic proliferation of filamentous Thiotrix, a sulfur-oxidizing bacterium commonly appearing under elevated organic loading (Levantesi et al. 2004, Nielsen et al. 2009). The filamentous growth of Thiotrix can reduce sludge compactness and settleability, temporarily decreasing the sludge volume index (SVI) and promoting partial washout of biomass, including polyphosphate-accumulating organisms, thereby impairing P removal (Chen et al. 2023). Importantly, these fluctuations primarily affect phosphorus removal, while carbon and nitrogen treatment remain largely stable due to the broader distribution and resilience of the microbial populations responsible for these processes (Seviour and Nielsen 2010).

**Figure 4:**
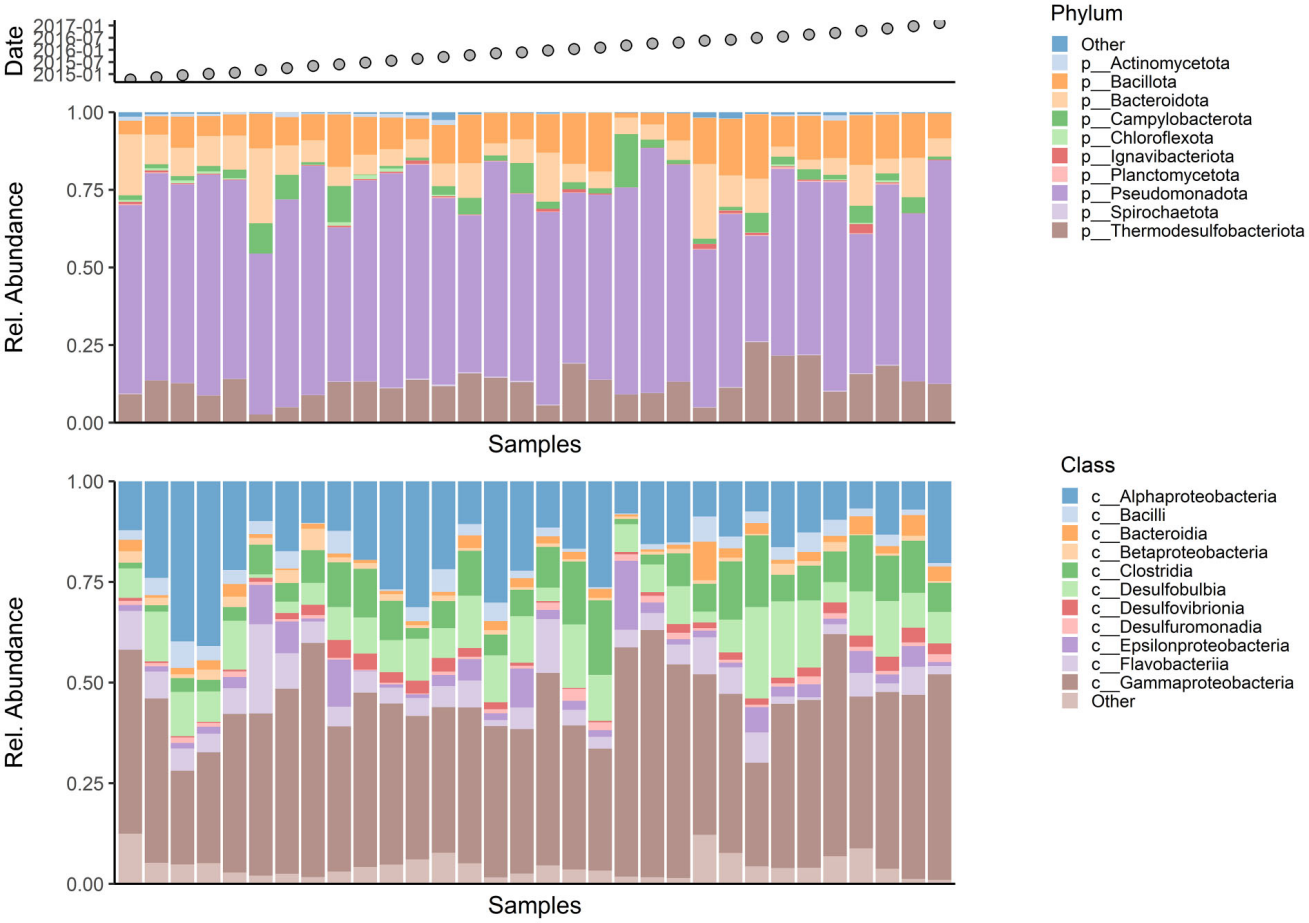
Composition barplot showing the relative abundances of the top 10 phyla and core classes of the activated sludge bacterial community within each sample. Taxonomic classifications are indicated by the color legend to the right, while the timeline legend at the top denotes the chronological order of sample collection.

Figure 4 presents the relative abundances of the top 10 most abundant phyla and core classes within the activated sludge bacterial community. The central role of the bacterial community in driving pollutant removal efficiency in activated sludge systems is well-documented, which has led to extensive investigations, particularly using 16S rRNA gene sequencing. The bacterial composition observed in this industrial saline wastewater treatment system is consistent with prior studies of activated sludge systems, featuring prominent phyla such as Pseudomonadota, Bacillota, Bacteroidota, Actinomycetota, and Chloroflexota (Wu et al. 2019, Begmatov et al. 2022). Notably, Pseudomonadota emerged as the most abundant phylum within this saline-activated sludge, aligning with established findings. Within this phylum, Gammaproteobacteria is the dominant class, known for its involvement in denitrification, organic matter degradation, and its capacity to thrive under varying environmental conditions (Kim et al. 2019, Wasmund et al. 2024).

Although typically underrepresented in domestic activated sludge systems, Thermodesulfobacteriota, including the sulfur-reducing class Desulfobulbia, were consistently detected as ribosomally active members of the microbial community throughout the monitoring period. Their enrichment is interpreted in a comparative ecological context relative to domestic wastewater treatment systems, which have been extensively characterized in the literature (Wu et al. 2019). The persistence of these taxa likely reflects environmental selection driven by the chemical characteristics of saline industrial influent, particularly elevated and temporally variable sulfate concentrations (Zhao et al. 2024). At the Oosterhorn saline wastewater treatment plant, sulfate concentrations exhibited substantial fluctuations, with a mean concentration of approximately 674.5 mg/l and a median of 112 mg/l, substantially exceeding typical levels reported for domestic wastewater. Such conditions are conducive to dissimilatory sulfate reduction under fluctuating redox regimes, supporting long-term niche specialization rather than a transient response to short-term influent perturbations.

At the class level, the core community consistently accounts for over 90% of the microbial composition, signifying a remarkably stable and specialized microbial ecosystem. This predominance underscores the critical roles of these taxa in sustaining ecosystem functionality, such as facilitating nutrient cycling and conferring resilience to environmental stressors. Their consistent representation reflects an adaptation to prevailing conditions and suggests minimal temporal variability. Such stability is often linked to efficient and predictable ecosystem performance, particularly in engineered systems like wastewater treatment processes.

**Figure 5:**
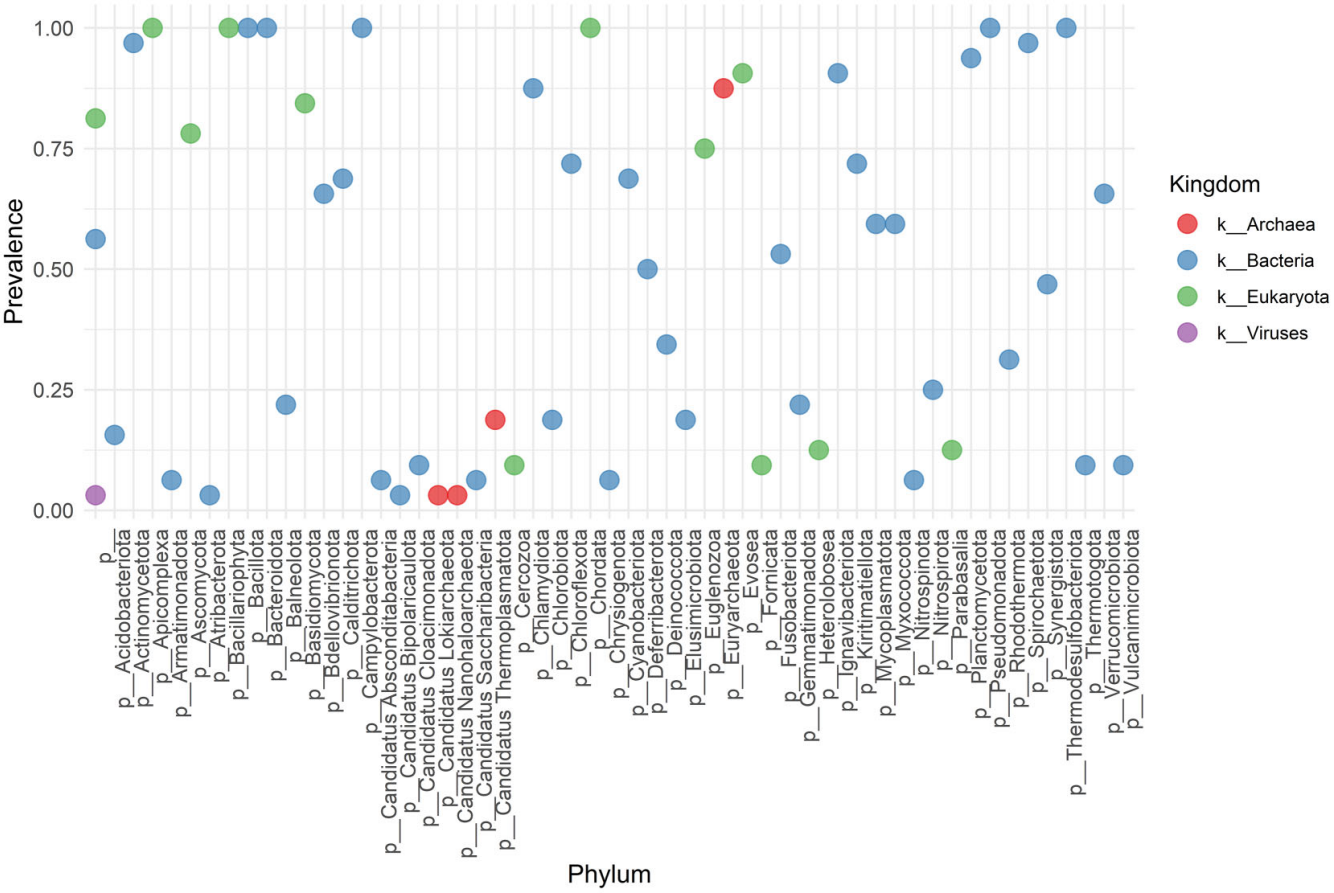
Prevalence plot of phylum-level abundances in the ribosomally active microbial community in alphabetical order, with coloring indicating the domain to which each phylum belongs. No arbitrary thresholds have been applied, allowing complete reproducibility of the analysis.

The structure of the ribosomally active microbial community treating the saline industrial wastewater in the plant is illustrated in Fig. 5. The prevalence plot depicts the distribution of various phyla across 32 samples, with coloring indicating the domain to which each phylum belongs. The core microbiome within the ribosomally active microbial community comprises predominantly bacterial and eukaryotic taxa, with notable bacterial contributions from Bacillota, Bacteroidota, Campylobacterota, Pseudomonadota, and Thermodesulfobacteriota.

**Figure 6:**
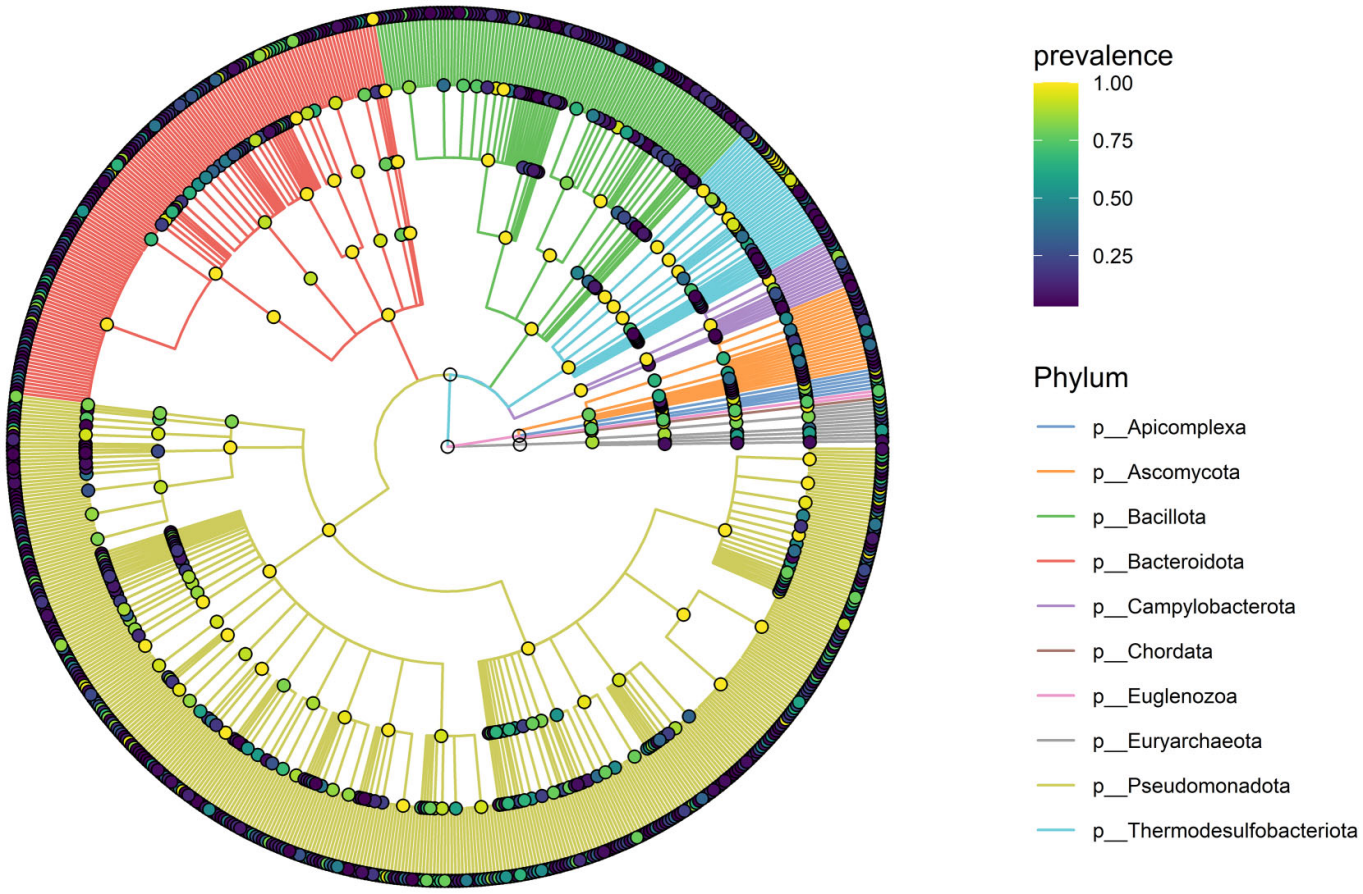
Prevalence tree of the top 10 most abundant phyla within the ribosomally active microbial community of the saline activated sludge system. Taxa with high prevalence across all 32 samples represent a core group of microorganisms that remained consistently ribosomally active throughout the study period. In contrast, taxa with low prevalence (≤ 0.25) likely represent specialized or transient members of the community that were ribosomally active only under specific environmental conditions.

The prevalence tree presented in Fig. 6 illustrates the prevalence of the top 10 most abundant phyla within the ribosomally active microbial community of the saline-activated sludge, extending to the genus rank for their respective subgroups.

The prevalence tree highlights greater diversity within bacterial phyla compared to eukaryotic counterparts, reflecting deeper taxonomic hierarchies at the class, order, and family levels. Subgroups within each phylum display differential prevalence; for example, while the phylum Pseudomonadota is present in all samples, its class Zetaproteobacteria occurs in 26 of 32 samples (81.25%). This pattern indicates that specific classes or genera, rather than entire phyla, are likely more relevant for ecosystem functioning and treatment performance in the activated sludge system.

### Functional redundancy

**Figure 7:**
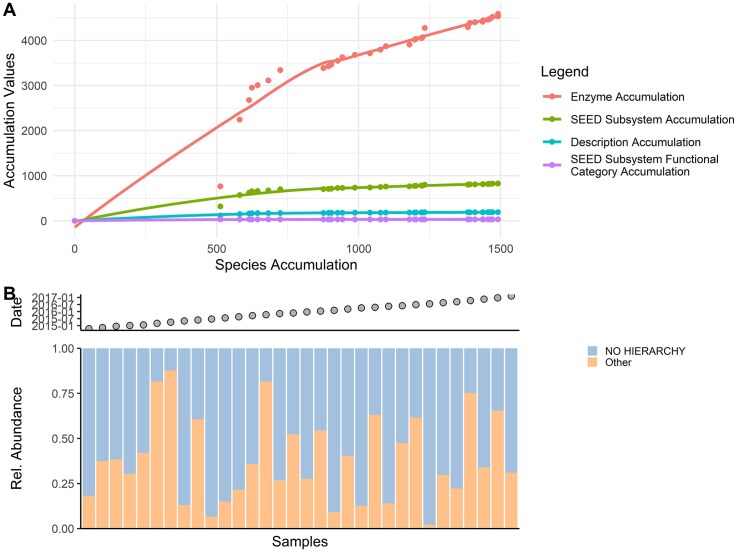
(A) Species-function accumulation curves using the collector method, which are fitted with curves generated using LOESS (Locally Weighted Scatterplot Smoothing) regression, capturing the accumulation dynamics of functional traits as species richness increases. (B) Composition bar-plot of functional genes in terms of characterized and non-characterized (NO HIERARCHY), with the timeline legend at the top denoting the chronological order of sample collection.

Figure 7 captures the accumulation dynamics of functional traits in relation to species richness, with curves fitted using LOESS (Locally Weighted Scatterplot Smoothing) regression.

The accumulation curves exhibit a characteristic biphasic pattern, marked initially by a steep rise in functional richness as each newly encountered species contributes distinct functional attributes. This early phase highlights the substantial role of taxonomic novelty in expanding the community’s functional repertoire. As species richness increases, the curves progressively plateau, signaling a saturation phase in which additional species contribute fewer novel functions, indicative of increasing functional redundancy. This pattern is consistently observed across both the collector method, which maintains the original sequence of sample acquisition to reflect the empirical sampling trajectory, and the randomized method, which generates an order-independent estimate by averaging accumulation across multiple permutations of sample order (Kindt and Oksanen 2019); the corresponding results are available in the project Git repository. Interpreted through the lens of Biodiversity–Ecosystem Function (BEF) theory, these trends suggest that while initial species additions are functionally enriching, subsequent additions primarily reinforce pre-existing functions, highlighting a threshold beyond which functional gains become marginal.

From the standpoint of redundancy and complementarity, the curves highlight two critical dynamics: complementarity, i.e. species contribute unique functions during the initial phase of rapid growth, and redundancy, where functions become increasingly shared among species as the curve approaches an asymptote. This high degree of functional redundancy within the ribosomally active microbial community of the activated sludge microbiome has profound ecological implications. Redundant species enhance the microbiome’s resilience, ensuring that essential ecosystem functions, such as nutrient cycling, organic compound degradation, or pathogen suppression, are maintained even in the face of disturbances, such as environmental fluctuations or exposure to antibiotics. This redundancy underpins the concept of ecological resilience, as overlapping functional roles provide a buffering capacity; if one species declines or is removed, others can compensate, preserving the stability and functionality of the system.

Functional redundancy in the saline activated sludge microbiome is evident from the rapid saturation of SEED subsystem accumulation curves, yet the diversity of specific enzymes continues to increase with additional species, albeit at a slower rate. This demonstrates that broad functions are redundant, while multiple enzymatic routes provide flexibility, underpinning the resilience of the microbial community.

However, it is important to acknowledge that this conclusion is indicative rather than definitive due to the presence of functional dark matter; a substantial group of functional genes annotated within the “No Hierarchy” category in SEED subsystems as shown in Fig. 7 B. These genes represent a poorly understood subset of the microbiome’s functional repertoire, likely arising from incomplete or limited databases that lack experimental validation or comprehensive annotation. The discovery, characterization, and inclusion of this functional dark matter in analyses could alter the observed plateau and extend the accumulation curves, delaying the apparent saturation of functional diversity.

### Functional diversity

**Figure 8:**
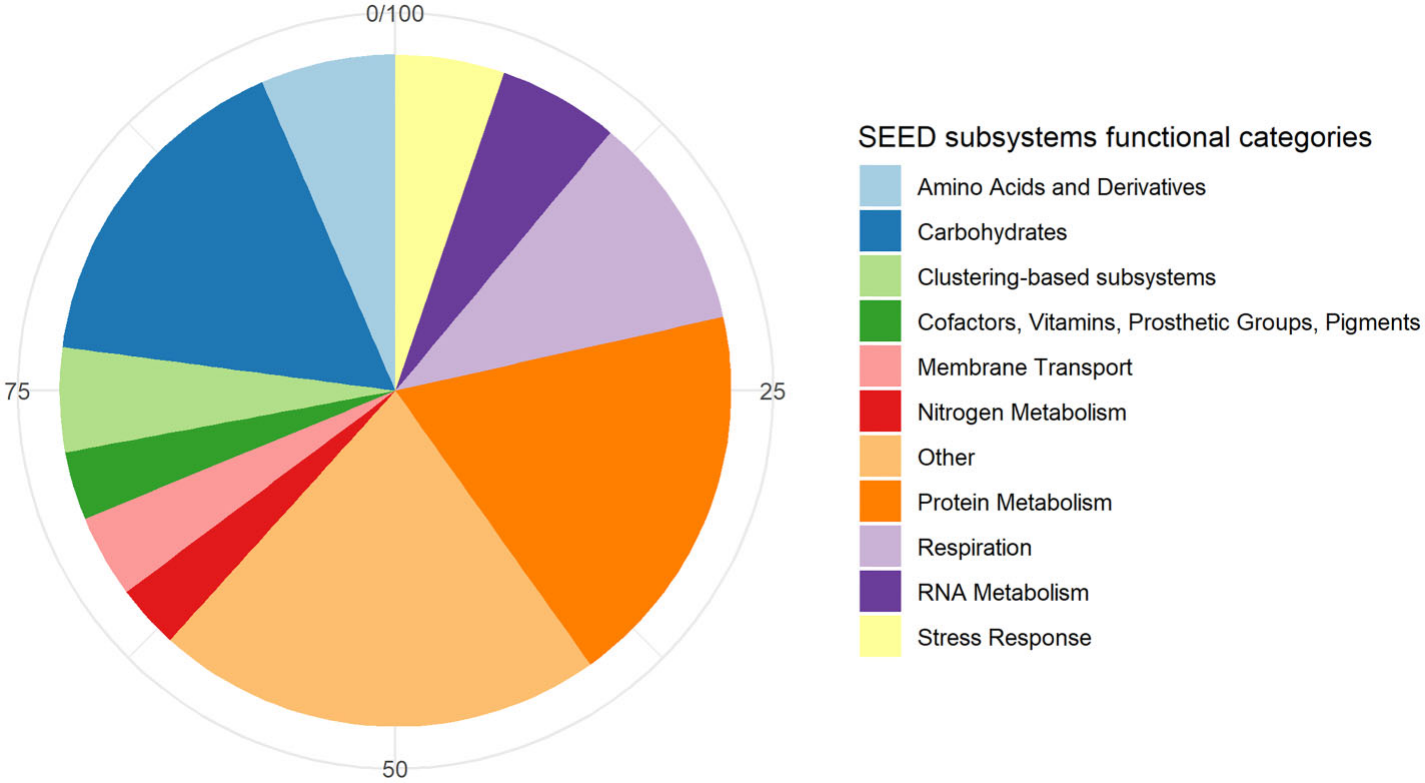
Distribution of the ten most abundant annotated SEED subsystem functional categories across 32 metatranscriptomic samples from the saline activated sludge system.

Analysis of the 32 metatranscriptomic samples revealed 31 distinct SEED subsystem functional categories, underscoring the extensive functional breadth of the saline activated sludge microbiome. Figure 8 presents a pie-chart representation of the ten most abundant annotated SEED subsystem functional categories aggregated across all metatranscriptomic datasets. These top ten categories collectively account for more than 75% of the total annotated transcript abundance detected across the system. This pronounced concentration of transcriptional activity within a limited subset of functional categories indicates that, although the community retains broad metabolic potential, its expressed metabolism is strongly structured around a core set of dominant physiological processes. Such functional skewness is consistent with ecological specialization driven by environmental filtering, whereby operational parameters, particularly cyclic aeration and elevated salinity, select for pathways central to reactor function. Specifically, dominant categories include those associated with energy generation (e.g. Carbohydrates and Respiration), nutrient turnover (e.g. Nitrogen Metabolism and Amino Acids and Derivatives), stress tolerance (e.g. Stress Response and Membrane Transport), and cellular maintenance and growth (e.g. RNA Metabolism, Protein Metabolism, and Cofactors, Vitamins, Prosthetic Groups, and Pigments). Together, these categories reflect active redox cycling, nitrogen transformation, osmoadaptation, macromolecular synthesis, and biosynthetic capacity within the system.

Importantly, the dominance of these functional groups does not imply reduced functional diversity. Rather, it reflects prioritization of energetically and ecologically central processes that sustain reactor performance and stability under saline and intermittently oxic conditions. Collectively, these patterns support a model in which the saline activated sludge microbiome is organized around a transcriptionally active core metabolism, buffered by auxiliary functions that confer robustness under dynamic operational constraints.

To explore associations between microbial taxonomy and functionality, we computed Spearman cross-correlations between phylum-level taxonomic abundances and SEED subsystems functional categories expression profiles, both CLR-transformed (centered log-ratio transformed) to account for the compositional nature of the data. The CLR transformation projects the data from the constrained simplex space into Euclidean space, enabling valid correlation analysis by mitigating spurious relationships inherent in relative abundance data (Chan and Li 2024). Spearman correlation, a non-parametric measure robust to non-normality and non-linearity, is well-suited for detecting monotonic associations in microbial communities (Francis and Sun 2024). Euclidean distance, a natural measure of similarity in Euclidean space, is used to quantify the proximity between samples based on their taxonomic and functional correlation patterns. Ward’s hierarchical clustering method, which minimizes within-cluster variance, is applied to group samples with similar taxonomic-functional relationships. This approach yields biologically and ecologically meaningful insights into taxon-function relationships and community structure, while addressing the constraints of compositional data (Martín-Fernández et al. 2024).

**Figure 9:**
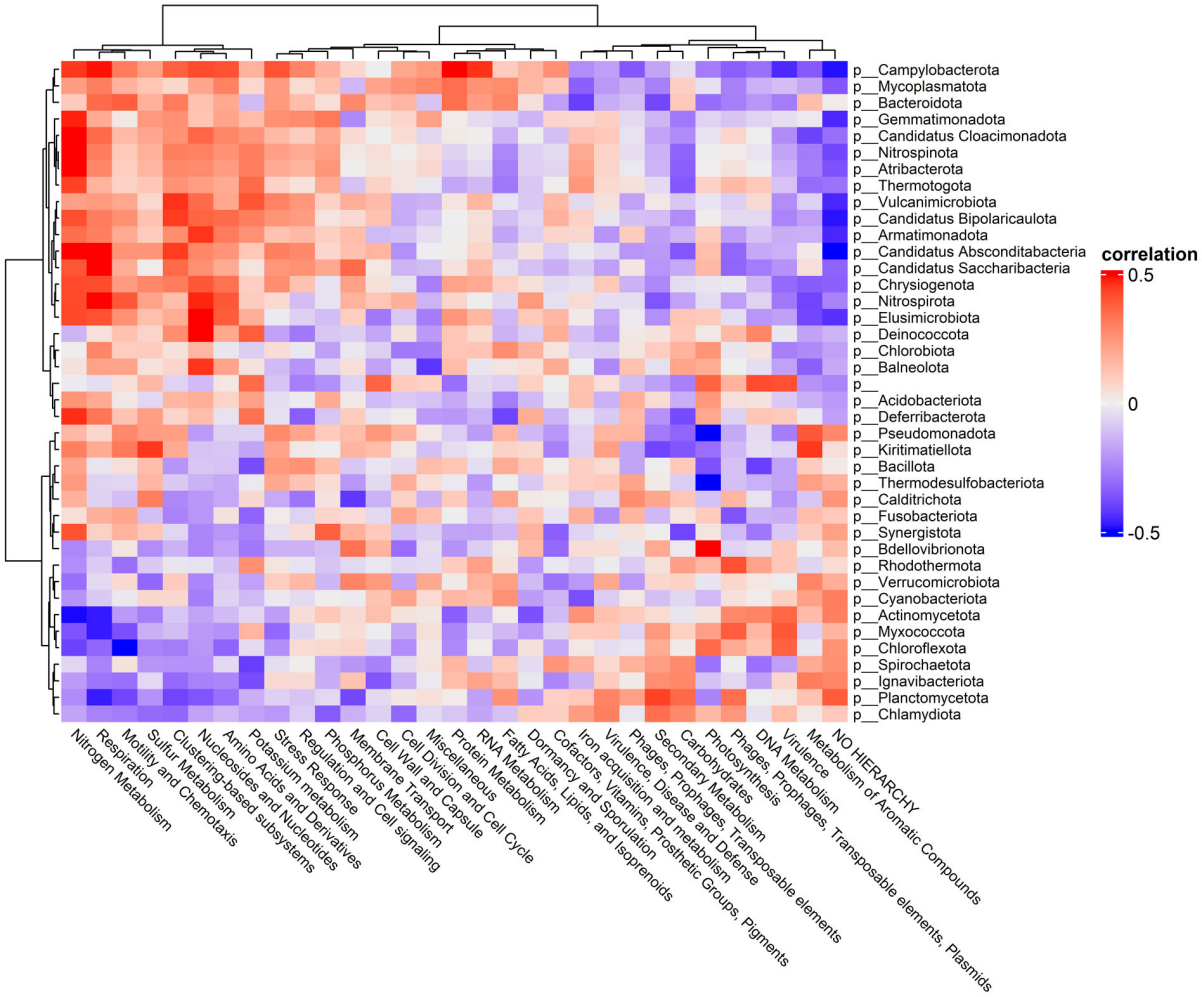
Cross-association heatmap of bacterial phyla and SEED subsystems functional categories. Cross-associations were calculated using Spearman correlation on CLR-transformed phylum-level abundances and SEED subsystems functional categories expression profiles, projecting compositional data into Euclidean space to mitigate spurious correlations. Euclidean distance was used to quantify similarity between samples, and Ward’s hierarchical clustering grouped samples with similar taxonomic-functional relationships, revealing biologically and ecologically meaningful associations between microbial taxa and their functional role.

The resulting cross-association heatmap presented in Fig. 9 reveals the complex relationships between bacterial phyla and functional diversity within the microbial community of the saline activated sludge. This analysis identifies two distinct clusters of functional categories alongside two primary clusters of bacterial phyla, providing insights into the ecological and operational dynamics within the facultative activated sludge reactor. The observed patterns reflect the intricate interplay between microbial community structure and metabolic processes, which are influenced by varying environmental and operational conditions such as oxygen availability.

The two functional clusters identified in the heatmap could correspond to the primary operational modes of the facultative activated sludge reactor, namely, aeration and no aeration phases, each shaped by fluctuating oxygen availability that governs microbial metabolic processes. The non-strict delineation of the two functional clusters in the cross-association heatmap highlights substantial intraspecific variability within SEED subsystem categories, indicating that enzymes within a given functional category can operate across a range of oxygen conditions.

**Figure 10:**
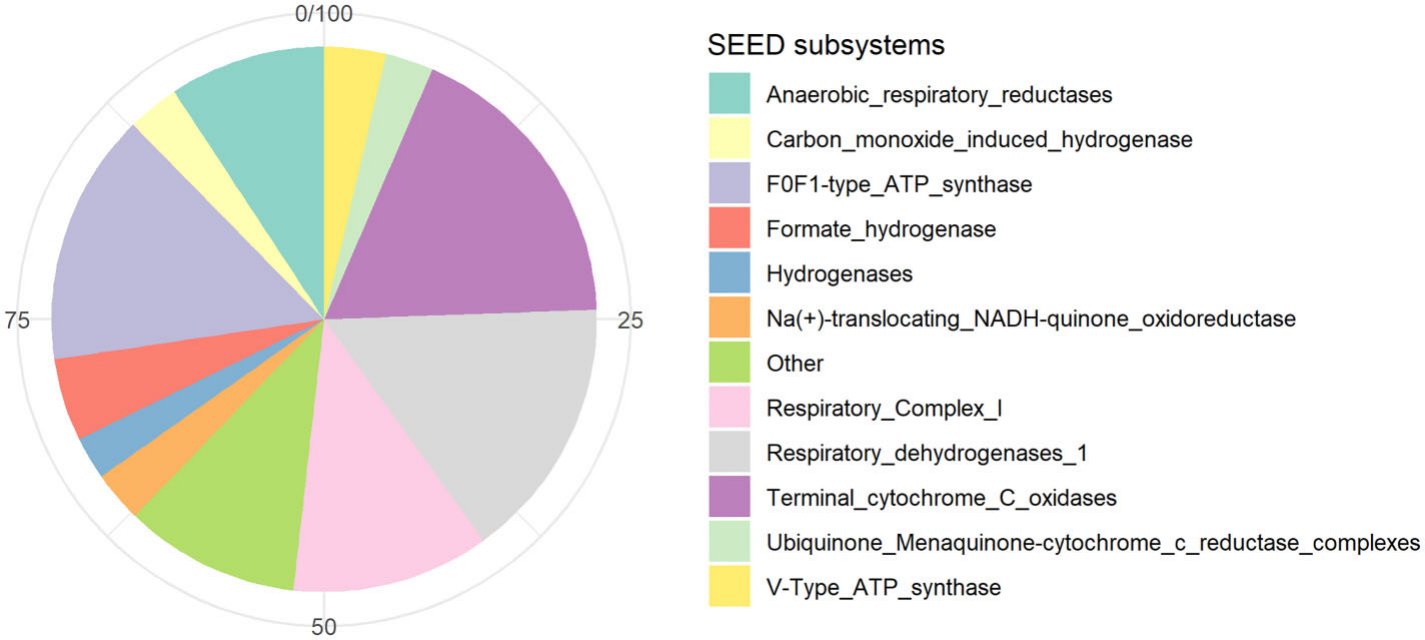
Relative distribution of SEED subsystem annotations within the respiration functional category.

Within the respiration category, both aerobic and anaerobic pathways coexist, reflecting functional differentiation under the dynamic environmental conditions of the facultative activated sludge reactor. Figure 10 presents a pie chart illustrating the distribution of SEED subsystems assigned to the respiration functional category, highlighting the relative contribution of the most transcriptionally abundant enzymes. The dominant respiration-related enzymes, collectively representing the top 90% of transcript abundance within this category, include terminal cytochrome c oxidases (17.9%), respiratory dehydrogenases 1 (15.6%), F0F1-type ATP synthase (15.0%), respiratory Complex I (11.8%), and anaerobic respiratory reductases (9.3%), along with lower-abundance contributors such as formate-hydrogenase (4.9%), hydrogenases (2.5%), carbon-monoxide induced hydrogenase (3.1%), and Na⁺-translocating NADH-quinone oxidoreductases (3.0%).

These highly expressed enzymes provide a mechanistically relevant view of the core energy-generating processes in the saline facultative activated sludge microbiome, capturing how the microbial community flexibly shifts between aerobic and anaerobic metabolism in response to dynamic aeration. Terminal cytochrome c oxidases and respiratory dehydrogenases primarily function under oxygen-rich conditions, whereas anaerobic respiratory reductases, formate-hydrogenase, and hydrogenases operate under anoxic or microaerophilic conditions, enabling energy conservation when oxygen is limited. Universal systems such as F0F1-type ATP synthase and V-type ATP synthase maintain cellular energy balance across both oxic and anoxic phases (Techtmann et al. 2011, Huang and Millar 2013). This metabolic flexibility underscores that functional genes are grouped based on their metabolic roles, rather than being strictly linked to specific environmental niches.

The two clusters of the microbial community indicated in the cross-association heatmap could reflect underlying differences in oxygen preferences and metabolic capabilities among bacterial phyla. One cluster could be the aerophilic cluster, which includes phyla that thrive in oxygen-rich environments. These phyla are primarily involved in aerobic processes, such as the degradation of organic matter and participation in nutrient cycling (Federation 2017). The second, the aerophobic cluster, consists of phyla adapted to oxygen-limited or anaerobic conditions. These organisms rely on anaerobic processes such as fermentation and anaerobic respiration to generate energy (de Beer et al. 1998). The presence of sub-clusters within both the aerophilic and aerophobic groups could reflect the microbial community’s ability to adapt to a range of environmental conditions, showcasing the ribosomally active microbial community’s adaptive versatility. Some phyla within the aerophilic cluster, for instance, can survive and function in transient anoxic conditions (Bueno et al. 2012). Similarly, certain taxa within the aerophobic cluster are facultative anaerobes, capable of surviving in both anaerobic and oxygen-limited environments (Pedraz et al. 2020). This metabolic plasticity and flexible adaptability are crucial for maintaining ecosystem stability, especially in environments like activated sludge reactors, where oxygen levels can fluctuate. In addition, the presence of sub-clusters within both aerophilic and aerophobic groups could also indicate the persistence of micro-niches within the facultative zones of activated sludge reactors across varying operational conditions. Gradients in oxygen, pH, and nutrient availability generate heterogeneous microenvironments, fostering niche specialization and promoting ecological diversity. Oxygen gradients, in particular, establish overlapping metabolic zones (Schramm, Santegoeds and Nielsen 1999), while the physical architecture of flocs provides microhabitats with stable conditions that facilitate the co-occurrence of diverse microbial populations (Nakaya, Jia and Satoh 2024). These micro-niches support functional redundancy and resilience in the face of fluctuating influent characteristics and operational regimes.

In addition, the non-rigorous delineation of the two clusters in the cross-association heatmap suggests that bacterial phyla encompass a spectrum of ecological strategies, ranging from generalists exhibiting broad metabolic flexibility capable of thriving under diverse environmental conditions to specialists with more stringent ecological preferences. Generalist phyla in activated sludge are characterized by their metabolic versatility, allowing them to thrive across aerobic, anoxic, and anaerobic conditions. These phyla possess genetic and enzymatic capabilities to switch between metabolic pathways depending on environmental parameters, such as oxygen availability. For example, members of phyla like *Pseudomonadota* often exhibit facultative metabolic strategies, enabling them to perform aerobic respiration in oxygen-rich zones and shift to anaerobic pathways in oxygen-depleted conditions (Arai 2011). This adaptability makes generalists indispensable for the functional resilience of the microbial community, particularly in fluctuating environments like facultative reactors. In contrast, obligate aerobes and obligate anaerobes represent specialists with narrow ecological niches. Obligate aerobes rely entirely on oxygen for energy generation and typically dominate in well-aerated zones of the reactor. Examples include certain members of the Nitrospinota (Kop et al. 2024). These phyla are unable to survive in anaerobic environments, as they lack the genetic machinery for alternative electron acceptor pathways. Obligate anaerobes, on the other hand, are highly adapted to oxygen-free environments. These organisms, such as some members of the Verrucomicrobiota and Spirochaetota, perform energy generation through anaerobic respiration or fermentation (Li et al. 2022). They are not active in aerobic conditions due to oxygen toxicity, which can disrupt their metabolic processes or damage cellular components. The coexistence of generalists and specialists reflects a division of labor within the ribosomally active microbial community, with generalists contributing to system-wide flexibility and specialists driving niche-specific processes (Wilson and Yoshimura 1994). This ecological stratification ensures that all available environmental niches are exploited, enhancing the overall efficiency and resilience of the activated sludge system.

Additionally, observed intra-phyla variability further supports the concept of functional and ecological plasticity. For instance, within Gammaproteobacteria, the genus Acinetobacter includes both strictly aerobic species (e.g. *A. calcoaceticus*) and facultative anaerobes (e.g. *A. johnsonii*), illustrating the diversity of respiratory strategies within a single lineage (Kim et al. 1997). This intraspecific heterogeneity enhances the microbiome’s ability to respond to shifts in environmental pressures and contributes to the robust performance of activated sludge systems. Overall, the clustering patterns identified in both functional and taxonomic dimensions are not strictly deterministic but are ecologically meaningful, capturing the adaptive structuring of microbial communities in response to fluctuating oxygen availability and reactor dynamics.

Notably, the core community, including Pseudomonadota, Thermodesulfobacteriota, Bacillota, Bacteroidota, and Campylobacterota, consistently accounts for over 90% of the microbial composition across all samplings in the facultative activated sludge reactor, despite fluctuating operational conditions. Their broad associations across multiple functional clusters in the heatmap highlight their functional versatility and ability to sustain key ecosystem processes under variable oxygen and physico-chemical conditions.

From the perspective of a community ecology framework (Vellend and Agrawal 2010), two key deterministic environmental selective dimensions shape this ecosystem: influent water quality and operational aeration regimes. The composition of the influent water represents a primary environmental filter, introducing a chemical composition that shapes microbial niche availability. Under these conditions, taxa with metabolic traits compatible with the prevailing chemical environment are preferentially selected, contributing to the emergence of stable core functional groups within the community.

Additionally, facultative activated sludge systems operate under alternating oxic and anoxic phases, generating periodic redox oscillations that structure microbial niches in time as well as in space. These cyclical transitions favor microorganisms with high metabolic flexibility, including the ability to switch between aerobic respiration and anaerobic respiratory pathways, and in some cases fermentation, depending on substrate availability and electron acceptor conditions.

Within this deterministic framework, stochastic processes such as immigration from the influent and ecological drift may also contribute to the persistence of low-abundance taxa and diversification of enzymatic pathways. The interplay between strong environmental selection and background stochasticity likely explains the observed pattern of functional redundancy at the SEED subsystem level, alongside continued diversification at the level of functional genes.

## Conclusion

Our longitudinal metatranscriptomic analysis of activated sludge microbiomes treating saline industrial wastewater reveals a dynamic yet functionally resilient microbial ecosystem shaped by strong environmental filtering. By capturing a broad range of community states over time in a full-scale, operationally complex system, this study resolves the persistent, metabolically active core microbiome and its expressed metabolic functions. We observed the emergence of *Thermodesulfobacteriota*, notably the sulfur-reducing class *Desulfobulbia*, as a consistent and functionally important member of this core community. This represents a divergence from the core communities typically observed in domestic wastewater systems, highlighting the selective pressures imposed by saline industrial influent.

Analysis of the relationship between taxonomic richness and functional gene richness across samples, demonstrated that functional potential saturates more quickly than species richness. This asymptotic pattern provides empirical evidence for functional redundancy: additional species contributed diminishingly to novel expressed functions, suggesting overlapping functional roles among taxa. However, the initial steep increase in functional richness with species richness also indicates functional complementarity, especially in low-diversity communities, where unique taxa contributed distinct functional capabilities.

The findings of this study highlight the strong potential of metatranscriptomics to advance our understanding of activated sludge microbiomes. Unlike 16S rRNA sequencing, which identifies bacterial taxa and only infers functional potential, particularly with full-length reads, without capturing gene expression, metatranscriptomics directly profiles actively transcribed genes across bacteria, archaea, and eukaryotes, providing a real-time view of microbial function. This approach allows for a comprehensive analysis of metabolically active genes driving ecosystem processes, offering new insights into microbial diversity, community adaptation, and overall ecosystem functionality within wastewater treatment systems.

Taken together, these findings support a model in which ecosystem functionality is maintained by a flexible community structure with overlapping roles (redundancy) and distinct contributions (complementarity), enabling robustness and adaptability under the selective pressures of industrial wastewater treatment.

## Data record

The 64 raw, unprocessed Illumina sequencing reads (fastq files) for all metatranscriptomes from the pair-end sequencing of the 32 samples have been deposited in the National Center for Biotechnology Information (NCBI) database under the Bioproject ID PRJNA1122484 (Mahajna 2024b) (http://identifiers.org/ncbi/BioProject:PRJNA1122484) and Sequence Read Archive (SRA) project accession number SRP513109 (Mahajna 2024c) (http://identifiers.org/ncbi/insdc.sra:SRP513109). The process data was uploaded to figshare (Mahajna 2024a) (https://doi.org/10.6084/m9.figshare.27073612.v1).
